# The effect on the equilibrium sickle cell allele frequency of the probable protection conferred by malaria and sickle cell gene against other infectious diseases

**DOI:** 10.1038/s41598-024-66289-2

**Published:** 2024-07-04

**Authors:** Farrokh Habibzadeh

**Affiliations:** Global Virus Network (GVN), Middle East Region, Shiraz, Iran

**Keywords:** Population genetics, Malaria, Sickle cell, Balanced polymorphism, Simulation, Population genetics, Computational models

## Abstract

If a mutated gene with heterozygous advantage against malaria, e.g., hemoglobin S (HbS) gene, is introduced in a small tribe, the gene (allele) frequency (*f*_*gene*_) increases until it reaches a steady state value (*f*_*eq*_) where the total mortality from malaria and sickle cell disease is a minimum. This is a classic example of balanced-polymorphism named malaria hypothesis. In a previous in silico study, assuming realistic initial conditions, it has been shown that the *f*_*eq*_ is around 14%, far less than the *f*_*gene*_ observed in certain parts of Africa, 24%. It seems that the malaria hypothesis, per se, could not explain such a high *f*_*gene*_, unless it is assumed that malaria and HbS gene can provide protection against other diseases. Using Monte-Carlo simulation, the current study was conducted to examine the effect on *f*_*eq*_ of five scenarios was examined. The studied scenarios consisted of different combinations of mortality of other diseases and the possible amounts of protections conferred by malaria and HbS gene against the diseases. Taking into account other diseases causing mortality in the population makes the *f*_*gene*_ rate of change steeper over generations. *f*_*eq*_ is an increasing function of the amount of protection conferred by HbS gene against other diseases. The effect of protection provided by malaria against other diseases on *f*_*eq*_, is however, variable—depending on the amount of protection conferred by HbS gene against other diseases, it may increase or decrease *f*_*eq*_. If malaria and HbS gene provide protections of 1.5-fold and threefold against other diseases, respectively, the *f*_*eq*_ is around 24%, the amount reported in certain tribes of Africa. Under certain scenarios, the *f*_*eq*_ attained is even higher.

## Introduction

Sickle-cell anemia is an example of autosomal recessive monogenic hereditary hemoglobinopathies. It is caused by a point mutation at the 6th position of the amino acid sequence of β-globin, where glutamic acid is substituted by valine^1^. The disease has a certain distribution across the globe, being highly prevalent in some places and extremely rare in other regions^2^. In certain parts of Africa, 40% of the population have sickle cell trait (heterozygous form of the disease)^3,4^; 4%, have sickle cell disease (homozygous form of the disease). This gives a gene (allele) frequency (*f*_*gene*_) of 24%^5–7^. With no treatment, most of children with sickle cell disease cannot survive to the reproductive age. Nevertheless, the *f*_*gene*_ has remained high over several decades^6,8^.

In 1946, Beet reported lower malarial infection rates among carriers of the sickle cell trait compared with non-sicklers^9^. Eight years later, Allison proposed that carriers of sickle cell gene are resistant to fatal falciparum malaria^3,7,10^. By the end of 1960’s, it was generally accepted that the high hemoglobin S (HbS) *f*_*gene*_ in certain parts of the world (e.g., Africa) is attributed to the advantage conferred by the HbS gene against malaria. This relationship became a classic example of “balanced polymorphism” in man, which is known as “malaria hypothesis”; *f*_*gene*_ for the advantageous heterozygous state increases until its incidence is balanced by the loss of homozygotes due to sickle cell disease complications^2^.

In a recent article, I have shown that to effectively provide protection against malaria within a short period, the HbS gene mutation needs to be happened in a small tribe with about 50 people at the reproductive age; the process would take more than 2000 years in a large population^11^. The *f*_*gene*_ increases until it reaches a steady state value (*f*_*eq*_). *f*_*gene*_ will, however, not constant thereafter; it fluctuates around *f*_*eq*_—a phenomenon termed “genetic drift”^12^. Under a realistic scenario, *f*_*eq*_ is around 14%^11^. *f*_*eq*_ rarely exceeds 15%, even in populations under intense malaria selection^13^. Even if the genetic drift is taken into account, the probability that the *f*_*gene*_ reaches the observed value of 24% or more, reported in certain tribes of Africa^5–7^, is very low (~ 0.005)^11^. *f*_*eq*_ could however reach the observed value of 24%, if the HbS gene and malaria confer protection against or reduce the mortality from other diseases prevalent in the region^11,14–18^. Using Monte-Carlo simulation, this in silico study was conducted to determine the effect of five scenarios on *f*_*eq*_. The scenarios consisted of different combinations of mortality rates of other diseases and the possible amounts of protections provided by malaria and the HbS gene against these diseases.

## Methods

### Monte-Carlo simulation

The methodology used in the current study was basically similar to that employed in the previous research^11^. In the Monte-Carlo simulation, the model parameters are considered stochastic or random variables; the technique involves running the model several times, each time using a set of input values randomly drawn from a set of possible values to determine various possible outcomes^19^. To have a valid realistic simulation, we need to identify the important variables and estimate their effects on the process.

#### Basic parameters

In a recent study, I examined the conditions under which the malaria hypothesis can best work^11^. Herein, five scenarios were investigated. All the scenarios were modifications of scenario 6 of the previous work^11^, where to adopt agricultural life, a tribe of 150 hunter-gatherers with 25 couples at reproductive age (the effective population size of 50) decided to settle nearby water where malaria and its associated conditions killed about 15% of their children before the reproductive age^11^. Such a transition from the hunter-gatherer to farmer life style happened around 4000–5000 years ago in western and Central Africa^20,21^.

#### Number of children

The average number of children of hunter-gatherers was around five for each couple^22^. With such spacing, parents could carry the youngest child, while the older children could walk and follow the tribe. Children were breast-fed for a longer period, which decreased the likelihood of another pregnancy^23^. More than 50% of the children died early before the reproductive age. In this way, the average number of five children for each couple kept the hunter-gatherers population size almost stationary^22^. In this simulation, variable number of children for each couple was assumed so that 10%, 15%, 50%, 15%, and 10% of the hunter-gatherer couples gave birth to 2, 3, 4, 5, and 6 children, respectively. This gives an average number of four children for each hunter-gatherer couple. With abundance of food after the hunter-gatherers settled and became farmer, they gave birth to more children, presumably an average of five children for each couple. Therefore, in the current simulation, it was assumed that from the 5th generation onward, when the transition from the hunter-gatherer to farmer population has completed, 10%, 15%, 50%, 15%, and 10% of the farmer couples gave birth to 3, 4, 5, 6, and 7 children, respectively^22^.

#### Population size

While the population size of the hunter-gatherers was almost stationary^22,24,25^, with abundance of food after a few generations, the number of children increased and the population of farmers grew. However, the population size did not grow indefinitely because of the limited resources available. It was assumed that the population grew until it reached a maximum of 6000 (an effective population size of 1000 couples)^11^. The growth was estimated by the following logistic function after the start of the growth (the 5th generation):

1 $$N_{t} = \frac{{1000N_{0} \, e^{0.15t} }}{{1000 + N_{0} (e^{0.15t} - 1)}},$$

where *N*_*0*_ and *N*_*t*_ represent the number of couples in reproductive age at the first five generations and *t* generations after the start of the growth, respectively. It was also assumed that there was cross-generational mating so that 5% of the parent population mated with offspring populations.

#### Other parameters

It was assumed that an advantageous mutated gene (e.g., HbS) occurred in one of the 50 people at the reproductive age, hence, a starting *f*_*gene*_ of 1%. While 85% of those homozygous for the gene (*SS* genotype) died of the disease complications before the reproductive age^26,27^, it was assumed that compared to normal people (*AA* genotype), gene carriers (*AS* and *SS* genotypes) conferred a tenfold protection against the fatal malaria^10,11,20,28^.

That was a brief description of scenario 6 in the previous study^11^. However, the scenarios studied herein were a little bit different; it was assumed that the malaria had a constant prevalence (*pr*_*m*_) of 40%, that children died of other diseases before the reproductive age with a cumulative probability (*M*_*O*_) of either 0% (scenario 1) or 25% (scenarios 2–5), and that malaria and HbS gene conferred protection against other diseases by a factor of *P*_*m,O*_ and *P*_*S,O*_, respectively, the amounts of which varied from scenario to scenario (Table 1). The cumulative mortality from malaria and its associated conditions before the reproductive age was kept at 15% for all scenarios (like scenario 6 of the previous study) so that the results were comparable^11^. In the current simulation, the protections provided by *AS* and *SS* genotypes against other diseases were considered equal.

**Table 1:** The initial values for the simulation in various scenarios studied.

Scenario	*M*_*O*_, %	*P*_*m,O*_	*P*_*S,O*_	*f*_*eq*_, %*
1^†^	0	1.0	1.0	13.9
2	25	1.0	1.0	13.9
3	25	1.5	1.0	14.7
4	25	1.0	3.0	25.7
5	25	1.5	3.0	24.7

For all scenarios it was assumed that the probability of death from malaria before the reproductive age was 15%; the protection against malaria conferred by *AS* and *SS* genotypes was 10, and that the probability of death from sickle cell disease (*SS* genotype) before the reproductive age was 85%. The protections conferred by *AS* and *SS* genotypes against other diseases were considered equal.

*M*_*O*_ mortality from other diseases, *P*_*m,O*_ protection conferred by malaria against other diseases, *P*_*S,O*_ protection conferred by *AS* or *SS* genotypes against other diseases, *f*_*eq*_ equilibrium gene (allele) frequency.

*Computed from Eqs. (2)–(4).

^†^Equivalent to scenario 6 in the previous article^11^.

The protection factor conferred against malaria by the *AS* and *SS* genotypes (*P*_*S,m*_), was assumed to be equal to 10 for both, like scenario 6 of the previous study^11^. The probabilities of death for each genotype are then:

2 $$\begin{aligned} M_{AA} = & \hspace{0.15cm} pr_{m} M_{m} + \left( {1 - pr_{m} M_{m} } \right)M_{O} , \\ M_{AS} = & \hspace{0.15cm} pr_{m} \left( {\frac{{M_{m} }}{{P_{S,m} }} + \frac{{M_{O} }}{{P_{S,O} {\mkern 1mu} P_{m,O} }} - \frac{{M_{m} M_{O} }}{{P_{S,m} {\mkern 1mu} P_{S,O} {\mkern 1mu} P_{m,O} }}} \right) + \left( {1 - pr_{m} } \right)\frac{{M_{O} }}{{P_{S,O} }}, \\ M_{SS} = & \hspace{0.15cm} M_{S} + \left( {1 - M_{S} } \right)M_{AS} , \\ \end{aligned}$$

where *M*_*x*_ designates the probability of death in a person with genotype *x*; *pr*_*m*_, the prevalence of malaria; *M*_*m*_, the probability of death from malaria (in this simulation, *pr*_*m*_ × *M*_*m*_ was assumed to be 15% so that the cumulative probability of death from malaria and its associated disorders before the reproductive age was kept constant so that the results of different scenarios were comparable); *M*_*O*_ and *M*_*S*_, the cumulative probabilities of death from other diseases and sickle cell disease (*SS* genotype) before the reproductive age, respectively; *P*_*S,m*_, the protection conferred by *AS* and *SS* genotypes against malaria, assumed to be 10 for both; and *P*_*S,O*_ and *P*_*m,O*_, the protections conferred the HbS gene carriers and malaria against other diseases, respectively. The fitness (*W* ) for *AS* and *SS* genotypes relative to *AA* genotype are then^11^:

3 $$\begin{aligned} W_{AA} = & \hspace{0.15cm} 1, \\ W_{AS} = & \hspace{0.15cm} \frac{{1 - M_{AS} }}{{1 - M_{AA} }}, \\ W_{SS} = & \hspace{0.15cm} \frac{{1 - M_{SS} }}{{1 - M_{AA} }}, \\ \end{aligned}$$

and

4 $$f_{eq} = \frac{{1 - W_{AS} }}{{1 - 2\,W_{AS} + W_{SS} }}.$$

#### Algorithm

The algorithm used in the study was basically similar to that employed in the previous article with minor modifications^11^. The pseudo-code of the simulation program used is shown in Table 2. In step 1, a 50-element array corresponding to a 50-person population (25 men and 25 women in reproductive age) was defined. The elements of the array reflected the genotype of the corresponding person (0 = *AA*, 1 = *AS*, 2 = *SS*). For the zeroth generation (the very first parent population), only one of the 50 persons was assumed to have the *AS* genotype.

**Table 2:** Pseudo-code of the simulation program.

1	Initialize the parent population with one heterozygous for the gene
	*Loop* for each generation from 0 to 100
2	Calculate *f*_*gene*_, *f*_*AS*_, *f*_*SS*_ /* zygotic level */
3	Selection process: eliminate those who died of malaria, sickle cell disease, or other diseases
4	Replace 5% of the population members with grandparents /* cross-generational mating */
5	Calculate *f*_*dead*_
6	Record the calculated frequencies
	* If* ((no homozygous *AND* no heterozygous) *OR* population size after selection < 2)
7	Record the generation at which “gene aborted” and *End*
8	Shuffle parent population array /* to provide a random mating */
	* Loop* for each couple alive in parent population
9	Determine the number of children and their genotypes based on Mendelian inheritance
	* Endloop*
10	Calculate the size of the next parent population /* growth */
11	Reinitialize the new parent population based on the offspring population
	*Endloop*

In step 2, *f*_*gene*_ and the frequencies of homozygous (*f*_*SS*_) and heterozygous (*f*_*AS*_) individuals in the population at the zygotic level were calculated. Then, 40% (*pr*_*m*_, the prevalence of malaria assumed in this study) of people randomly selected from the population were supposed to have malaria.

In step 3, based on the genotype, presence or absence of malaria in each person in the population, and the amounts of protections conferred by HbS gene against malaria and the protections provided by malaria and HbS against other diseases, the probability of death before the reproductive age from either sickle cell disease, malaria, or other diseases was computed for each of the population members.

In step 4 of the simulation, 5% of the parent population was replaced by the members selected at random from the grandparent population (cross-generational mating).

In step 5, the frequency of parent population who died was calculated. All the calculated frequencies (steps 2 and 5) were then recorded for further analysis (step 6). In step 7, it was checked if any gene carriers (*AS* or *SS* genotypes) still remained in the parent population after steps 3 and 4. If no HbS gene carriers remained or if the number of people in the parent population was less than two, it was concluded that the HbS gene was aborted and the generation at which it happened was recorded; else, the program proceeded to the next step.

In step 8, to give an equal chance to each survivor in the parent population to marry another, using a pseudo-random generator algorithm^29^, the population array elements were shuffled.

In step 9, those of the parent population corresponding to even positions of the array (0, 2, 4, etc.) mated with those corresponding to their next element (positions 1, 3, 5, etc.) in the array to produce children according to their genotypes and Mendelian inheritance; the genotype of each child was then determined. The number of children for each couple was determined at random from a lookup table determining the number of children and its probability for each couple depending on whether they were hunter-gatherer or farmer.

In step 10, the parent population size of the next generation was computed. In step 11, the members of the new parent population were selected at random from the offspring population. The whole process repeated from step 2 for 100 generations (~ 2500 years).

To eliminate the chaotic effects caused by inherent randomness of the Monte-Carlo method^19^, the arithmetic mean and the standard deviation (SD) of the values obtained from 10,000 consecutive repeats of the program was calculated. For each scenario, the mean was taken as the final refined result in each generation; the mean ± 1.96 × SD, the 95% confidence interval around the mean in each generation.

### Scenarios

Five scenarios were studied (Table 1). Assuming that other diseases had no mortality, scenario 1 was equivalent to scenario 6 described in the previous article^11^. Scenario 2 was similar to scenario 1, except that each person in each generation ran a 25% risk (*M*_*O*_) of death from other diseases (in addition to malaria and sickle cell disease), but neither malaria nor the HbS gene conferred protection against other diseases. Scenario 3 was similar to scenario 2, except that only malaria conferred 1.5-fold protection against other diseases (*P*_*m,O*_). Scenario 4 was similar to scenario 2, except that only the HbS gene conferred threefold protection against other diseases (*P*_*S,O*_). Scenario 5 was similar to scenario 2, except that both malaria and the HbS gene conferred 1.5-fold and threefold protection against other diseases (Table 1). In all scenarios, it was assumed that compared with those with *AA* genotype, gene carriers (*AS* and *SS* genotypes) provided a tenfold protection against malaria (*P*_*S,m*_). It was also assumed that the cumulative probability of death from malaria and its associated disorders before the reproductive age was 15%.

### Ethics

This in silico study did not involve any humans or animals or their tissue samples. Therefore, no institutional review board approval was necessary.

## Results

**Figure 1:**
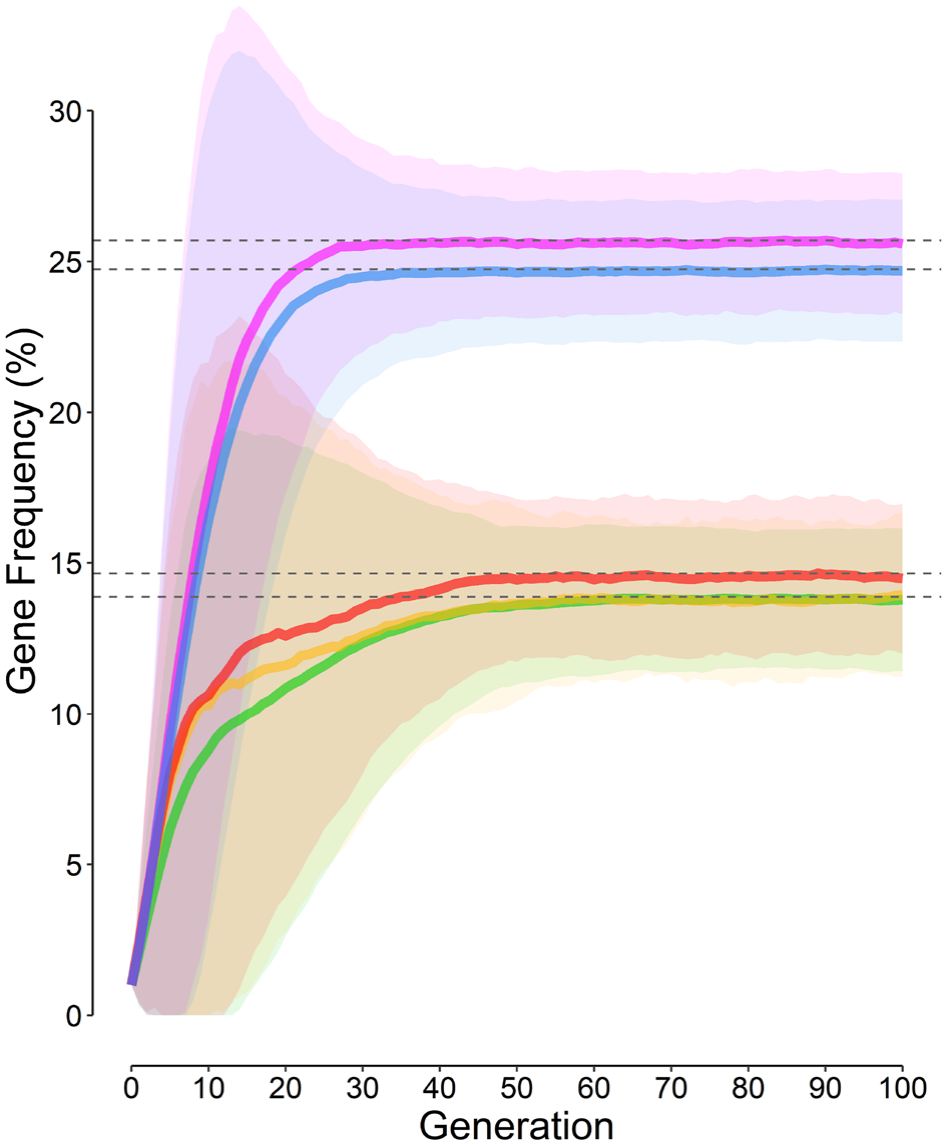
Variation of the gene (allele) frequency (*f*_*gene*_) over generations. The value at each point on each curve is the mean value of the *f*_*gene*_ at each generation obtained from 10,000 repeats of the corresponding scenario. Green curve is the *f*_*gene*_ under scenario 1 (Table 1, scenario 6 of the previous article^11^) where the mortality of other diseases was considered zero; yellow curve, scenario 2, where although other diseases killed 25% of people before the reproductive age, neither malaria nor the hemoglobin S (HbS) gene conferred protection against other diseases; red curve, scenario 3 (only malaria protection); magenta curve, scenario 4 (only HbS gene protection); and blue curve, scenario 5 (malaria and HbS gene protections). The shaded regions are the 95% confidence intervals for the *f*_*gene*_ under scenarios studied. The horizontal dashed gray lines represent the equilibrium *f*_*gene*_ computed from Eq. 4 (Table 1). The *f*_*gene*_ in scenarios 1 and 2 are similar to that has been reported for scenario 6 of the previous article^11^.

Under all scenarios studied (Table 1), *f*_*gene*_ increased over generations and reached a plateau, the *f*_*eq*_. Scenarios merely differed by *f*_*eq*_ and the rate of change of *f*_*gene*_ (Fig. 1). The *f*_*eq*_ values obtained by the simulation (Fig. 1, horizontal dashed gray lines) were very similar to those computed by Eq. (4) (Table 1).

### Scenario 1

Given that other diseases did not cause any mortality, scenario 1 was very similar to scenario 6 of the previous study (Fig. 1, green curve)^11^; no surprise the *f*_*eq*_ was 13.9% (Fig. 1), very similar to what has been reported in the previous study^11^.

### Scenario 2

Here, the cumulative probability of death from other diseases before the reproductive age (*M*_*O*_) was 25%. Although people died of other diseases, neither malaria nor the HbS gene conferred protection against other diseases. The course of the curve (Fig. 1, yellow curve) was different from that in scenario 1 (Fig. 1, green curve), but the *f*_*eq*_ did not differ from that in scenario 1 (Table 1).

### Scenario 3

This scenario was similar to scenario 2, except that malaria conferred 1.5-fold protection against other diseases, but no protection was provided by the HbS gene against other diseases; the *f*_*eq*_ was a little bit more than that observed for scenarios 1 and 2 (Table 1).

### Scenario 4

This scenario was similar to scenario 2, except that the HbS gene conferred threefold protection against other diseases, but no protection was provided by malaria against other diseases; the *f*_*eq*_ was almost 26% (Fig. 1, magenta curve).

### Scenario 5

The scenario was a combination of scenarios 3 and 4—malaria provided 1.5-fold and the HbS gene conferred threefold protection against other diseases. The *f*_*eq*_ was almost 25%, lower than that in scenario 4 (Fig. 1, blue curve).

### The equilibrium gene (Allele) frequency

**Figure 2:**
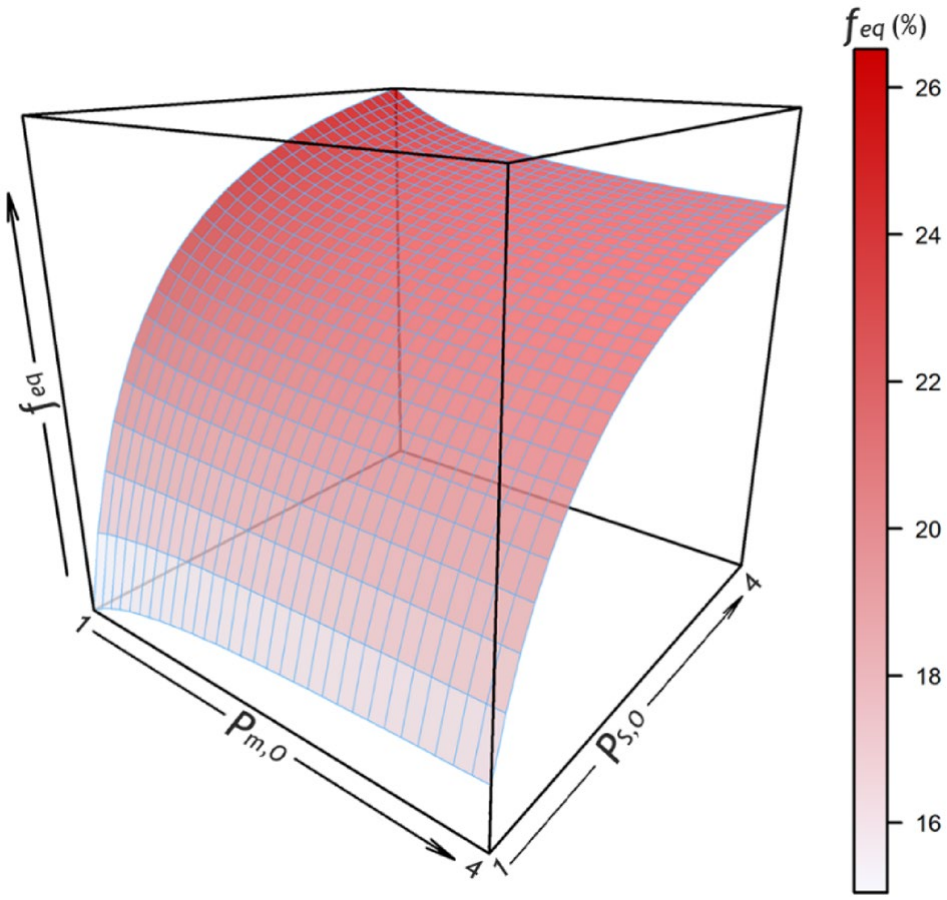
Equilibrium gene (allele) frequency (*f*_*eq*_) for different values of protections conferred by malaria (*P*_*m,O*_) and the hemoglobin S (HbS) gene (*P*_*S,O*_) against other diseases. *f*_*eq*_ is a minimum when neither malaria nor the HbS gene conferred protection (*P*_*m,O*_ = *P*_*S,O*_ = 1); it is a maximum when *P*_*m,O*_ = 1 and *P*_*S,O*_ = 4.

*F*_*eq*_ depends on both *P*_*m,O*_ and *P*_*S,O*_ (Fig. 2). However, when ∂*f*_*eq*_ / ∂*P*_*m,O*_ vanishes, *f*_*eq*_ is independent of *P*_*m,O*_, which is when:

5 $$P_{S,O}^{*} = \frac{{M_{O} \,M_{m} \left( {P_{S,m} - 1} \right)\left( {pr_{m} - 1} \right) + M_{m}^{2} \,pr_{m} - M_{m} \left( {P_{S,m} \,pr_{m} + 1} \right) + P_{S,m} }}{{M_{m}^{2} \,pr_{m} - M_{m} \left( {P_{S,m} + pr_{m} } \right) + P_{S,m} }}.$$

**Figure 3:**
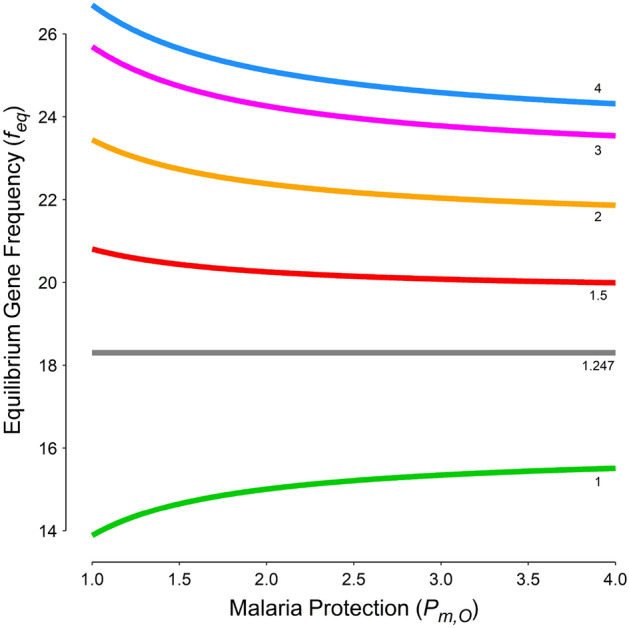
The equilibrium gene (allele) frequency (*f*_*eq*_) against the amount of protection conferred by malaria against other diseases (*P*_*m,O*_) for different levels of protection provided by the hemoglobin S (HbS) gene. Figures near the curves are the amount of protections provided by the HbS gene against other diseases (*P*_*S,O*_). There is a certain value for *P*_*S,O*_ (Eq. 5) where the *f*_*eq*_ is constant regardless of *P*_*m,O*_ (horizontal gray line).

**Figure 4:**
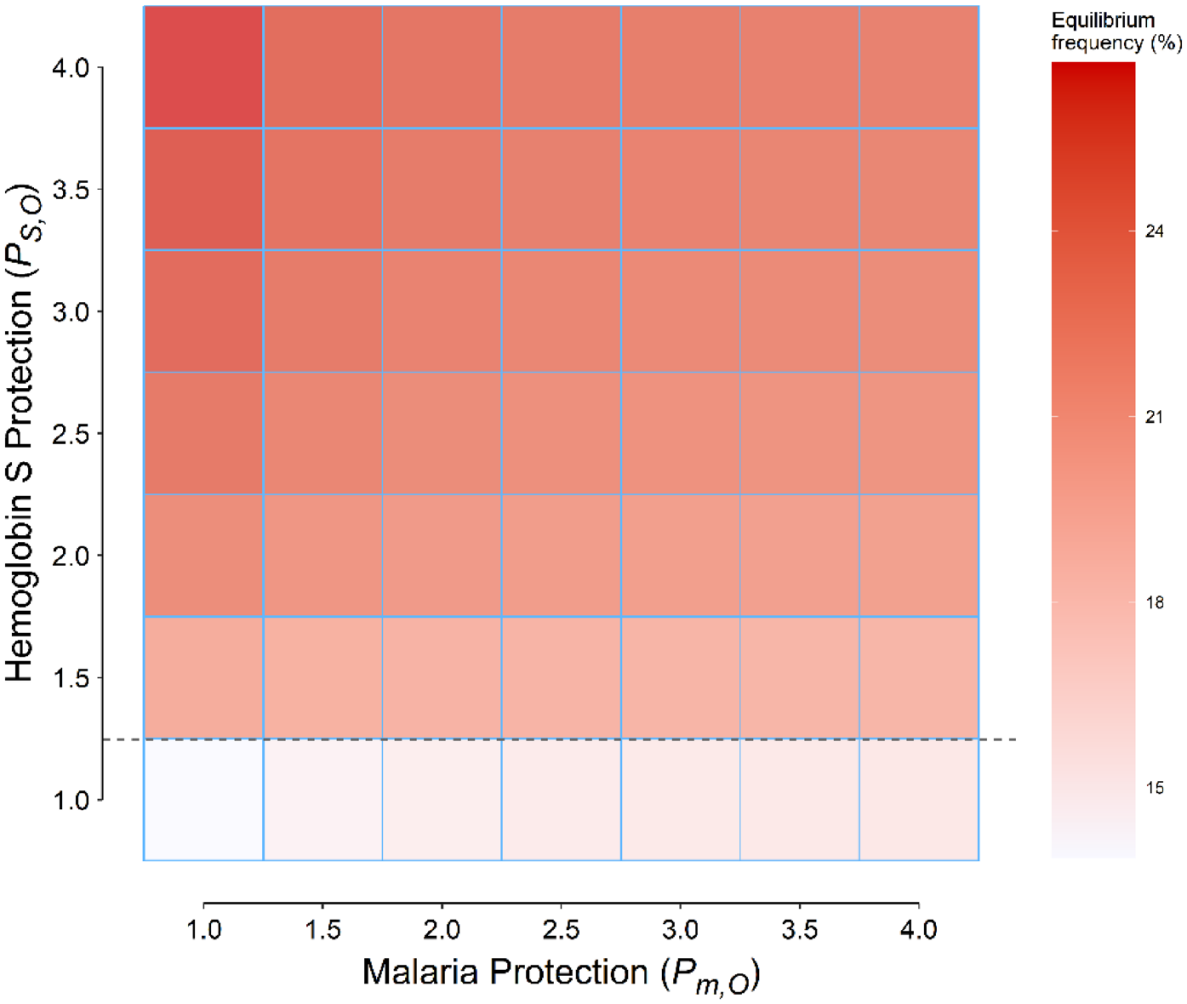
Equilibrium gene (allele) frequency (*f*_*eq*_) for different combinations of protections conferred by malaria and the hemoglobin S (HbS) gene against other diseases. The minimum *f*_*eq*_ corresponds to a point where neither malaria nor the HbS gene conferred protection, the white tile (*P*_*m,O*_ = *P*_*S,O*_ = 1); the maximum, where the protection conferred by malaria is nil (*P*_*m,O*_ = 1), but the HbS gene provided the highest protection (*P*_*S,O*_ = 4). The horizontal dashed line corresponds to the value derived from Eq. (5), 1.25; with *P*_*S,O*_ < 1.25 (the first row at the bottom), *f*_*eq*_ is an increasing function of *P*_*m,O*_; for *P*_*S,O*_ > 1.25 (other rows), it is decreasing.

Plugging in the values used for this simulation, the $P_{S,O}^{*}$ is almost 1.25; *f*_*eq*_ is stationary for all values of *P*_*m,O*_ (Fig. 3, horizontal gray line). For *P*_*S,O*_ less than this value, *f*_*eq*_ is an increasing function of *P*_*m,O*_ (Fig. 3, green curve); otherwise, it is decreasing (Fig. 3). *f*_*eq*_ is an increasing function of *P*_*S,O*_ for all values of *P*_*m,O*_ (Figs. 2, 4).

## Discussion

The results of scenario 1, where there was no mortality from other diseases, were expectedly similar to scenario 6 of the previous study^11^. In scenario 2, the cumulative probability of death from other diseases before the reproductive age was 25%, but neither malaria nor HbS gene conferred protection against other diseases. Therefore, as expected, the *f*_*eq*_ was the same as that in scenario 1; the only determinants of *f*_*eq*_ were the protection provided by *AS* and *SS* genotypes against malaria. Presence of a disease with high mortality increased the rate of change of *f*_*gene*_ over generations (Fig. 1, all but the green curve).

Certain disease conditions may provide protection against other diseases or decrease their mortality and morbidity rates. For instance, it has been shown that α^+^-thalassemia confer protection against malaria as well as other infectious diseases^30^. A cohort study has shown that the presence of *AS* genotype is associated with a decreased all-cause mortality among young children^14^. A recent systematic review revealed that sickle cell disease confers protection against human immunodeficiency virus (HIV) infection^16^. Sickle cell disease can also provide protection against the intra-erythrocytic parasite, *Babesia*^17^. Presence of HbS has also been shown to be associated with a lower mortality and morbidity rate from severe acute respiratory syndrome coronavirus 2 (SARS-CoV-2) infection^18^. The non-specific protection does not limited to hemoglobinopathies. An ecological study shows the association between the prevalence of malaria and the cumulative incidence of SARS-CoV-2 infection, an observation that is biologically plausible^15^. The plasmodium double-stranded DNA and hemozoin can trigger Toll-like receptor 7 with resultant activation of intracellular downstream signaling cascade reactions leading to production of type I interferons and pro-inflammatory cytokines resulting in short-term non-specific protection against other infectious diseases^31,32^. Therefore, it seems that both malaria and HbS gene may confer non-specific protection against other diseases.

Protection conferred by the HbS gene (*P*_*S,O*_ > 1) should expectedly increase *f*_*eq*_^11^; this is in keeping with the results of the current study too (Figs. 2, 4). This is why the *f*_*eq*_ in scenario 4 (*P*_*S,O*_ = 3, *P*_*m,O*_ = 1 [no protection]) is 25.7% (Fig. 1, magenta curve), which can explain the high *f*_*gene*_ reported from certain African tribes^3,4^, *QED* (*quod erat demonstrandum*).

The protection provided by malaria is a little bit tricky. If malaria also conferred protection, such as what assumed in scenario 5 (*P*_*S,O*_ = 3, *P*_*m,O*_ = 1.5), the *f*_*eq*_ decreased by 1% compared to scenario 4. At the first glance, this might seem reasonable; the protection conferred by malaria against other diseases should diminish the advantage of HbS gene against malaria; the *f*_*eq*_ should thus decrease, as what was observed in scenario 5 compared to scenario 4. But, things are not as simple as they seem; *f*_*eq*_ depends on the amount of protections conferred by malaria and the HbS gene. If the protection provided by the HbS gene (*P*_*S,O*_) is less than a certain value,$P_{S,O}^{*}$ (Eq. 5), *f*_*eq*_ is an increasing function of *P*_*m,O*_ (Figs. 3, 4); malaria protects more people against other diseases than it kills. As a consequence, to protect people against malaria, *f*_*gene*_ increases and a new equilibrium state will be attained. In scenario 3, the protection conferred by malaria was 1.5-fold whereas the HbS gene did not protect at all; compared to scenario 2, *f*_*eq*_ slightly increased to 14.7% (Fig. 1, red curve). If malaria and the HbS gene could confer protection against other diseases, the main determinant of *f*_*eq*_ is the total mortality rate caused by malaria and its related disorders, sickle cell disease, and other diseases; things are changed toward a situation where the rate is a minimum. Malaria should not always be considered an evil. If the protection provided by the HbS gene is high enough (> $P_{S,O}^{*}$), the above-mentioned argument does no longer hold; the protection provided by the HbS gene against other diseases outweighs the beneficial effect of protective malaria; *f*_*eq*_ becomes a decreasing function of *P*_*m,O*_ (Figs. 3, 4).

Taking into account other diseases associated with mortality results in a steeper *f*_*gene*_ rate of change over generations. This makes the expected protection predicted by malaria hypothesis occurs within a period shorter than that would occur without considering the mortality of other diseases (Fig. 1, scenarios 2–5 as compared to scenario 1); under scenario 5 (Fig. 1, blue curve), *f*_*gene*_ reached the *f*_*eq*_ of 24.7% after almost 25 generations (~ 600 years) compared to 45 generations (~ 1000 years) under scenario 1 (Fig. 1, green curve).

People infected with malaria are at higher risk of bacteremia and its associated mortality^33^. One may assert that the higher malaria-associated infectious disease mortality could lead to higher *f*_*eq*_. However, the cumulative probability of 15% taken into account for the malaria death in the current study included all direct and indirect mortalities; a value compatible with many in silico studies so far conducted^34–36^.

In conclusion, to better understand the malaria hypothesis, we need to take into account the probable protections conferred by malaria and the HbS gene against other diseases too. If malaria confers protection against other diseases, the *f*_*eq*_ may ironically increase under certain circumstances. To get a more precise picture of what really happens, protections provided by other hemoglobinopathies (e.g., thalassemia, hemoglobin C) and glucose-6-phosphate dehydrogenase (G6PD) deficiency could also be considered in future simulations.

## Data Availability

The pseudo-code of the simulation program is presented in the manuscript; the source code developed in *C* programming language will be available on request from the corresponding author.
